# Beyond Acid Suppression: Mechanistic and Translational Perspectives on the Pleiotropic Pharmacology of Pantoprazole

**DOI:** 10.3390/biology15211937

**Published:** 2026-10-25

**Authors:** Shakta Mani Satyam, Manasvi Udaya Kumar, Ebrahim Safaii, Shams Al-dawoodi, Ilmia Shameer, Rashmi Kumari, Abdul Rehman, Mohamed El-Tanani, Afnan Afroz, Anuradha Asokan Nair, Karthik Nair, Murk Jai Pal Paryani

**Affiliations:** 1 Department of Pharmacology, RAK College of Medical Sciences, RAK Medical and Health Sciences University, Ras Al Khaimah 11172, United Arab Emirates; 2 A. J. Institute of Hospital Management, A.J. Hospital and Research Center, Mangalore 575004, India; 3 Department of Pathological Sciences, College of Medicine, Ajman University, Ajman 346, United Arab Emirates; 4 RAK College of Pharmacy, RAK Medical and Health Sciences University, Ras Al Khaimah 11172, United Arab Emirates

**Keywords:** pantoprazole, proton pump inhibitors, pleiotropic pharmacology, proton homeostasis, molecular mechanisms, drug repurposing, translational pharmacology

## Abstract

Pantoprazole is widely used for the management of acid-related gastrointestinal disorders through inhibition of the gastric H^+^/K^+^-ATPase. Beyond its established acid-suppressive action, experimental studies have investigated its potential effects on cellular proton homeostasis, autophagy, oxidative and inflammatory signaling, cancer biology, antimicrobial resistance, glucose regulation, neurological responses, hepatic fibrosis, and iron-related outcomes. This narrative review critically examines these proposed biological effects and distinguishes established pharmacology from experimental mechanisms, preclinical findings, and human clinical evidence. Particular attention is given to the relationship between experimental concentrations and human exposure, the acid-dependent activation of pantoprazole, the distinction between direct molecular targets and downstream cellular responses, and the challenges of translating laboratory findings into clinical benefit. Pantoprazole also has a comparatively favorable CYP2C19-mediated drug-interaction profile relative to omeprazole and esomeprazole, although this advantage does not imply the absence of all clinically relevant interactions. The evidence across proposed repurposing indications remains heterogeneous, and biological activity in experimental models should not be interpreted as established therapeutic efficacy. Further exposure-informed mechanistic studies, pharmacokinetic and pharmacodynamic validation, and appropriately designed clinical trials are required to determine which proposed effects warrant clinical development. Pantoprazole should therefore be regarded as a candidate for mechanism-informed drug-repurposing research rather than an established treatment for the non-gastrointestinal indications discussed in this review.

## Simple Summary

Pantoprazole is a commonly used medicine that reduces stomach acid and is prescribed for conditions such as acid reflux and peptic ulcers. Researchers have also investigated whether it affects other biological processes, including inflammation, cellular stress, cancer-cell responses, bacterial resistance, glucose regulation, and tissue injury. This review examines those findings and explains why results from laboratory experiments do not necessarily predict benefits in patients. Pantoprazole has a comparatively favorable drug-interaction profile relative to some other proton pump inhibitors, but this does not mean that it has no interactions or that it is proven to treat diseases beyond its established indications. The concentrations used in experiments, the way pantoprazole is activated, and the quality of the available evidence are all important when evaluating its potential for repurposing. More rigorous studies are needed to determine whether the proposed effects can be achieved safely and effectively in humans.

## 1. Introduction

Proton pump inhibitors (PPIs) are substituted benzimidazole derivatives widely used in the management of acid-related gastrointestinal disorders. Their principal pharmacological action is inhibition of the gastric hydrogen/potassium adenosine triphosphatase (H^+^/K^+^-ATPase), the final common pathway of gastric acid secretion. Although PPIs share this primary mechanism, they differ in their pharmacokinetic properties, metabolic pathways, and potential for drug–drug interactions. Pantoprazole is a clinically established PPI used for conditions including peptic ulcer disease and gastroesophageal reflux disease. A relevant comparative pharmacological characteristic of pantoprazole is its relatively low propensity for clinically important CYP2C19-mediated interactions compared with omeprazole and esomeprazole [1,2]. This characteristic can be useful when selecting acid-suppressive therapy for patients receiving multiple medications. Within the secretory canaliculi of gastric parietal cells, pantoprazole is activated under acidic conditions to form its active intermediate, which then covalently modifies the gastric H^+^/K^+^-ATPase, producing prolonged inhibition of gastric acid secretion. PPIs are generally more effective acid suppressors than drugs acting at earlier steps of the gastric acid secretion pathway. They are widely used to treat acid-related disorders and to prevent or manage upper gastrointestinal complications. An extensive body of literature describes the pharmacokinetics, pharmacodynamics, therapeutic effects, and safety of pantoprazole in humans [3,4,5,6]. Although its established clinical use, characterized pharmacology, and comparatively favorable interaction profile provide a rationale for investigating pantoprazole as a drug-repurposing candidate, these properties do not establish that it has superior non-gastric activity or therapeutic efficacy compared with other PPIs. Accordingly, its proposed repurposing effects require compound-specific evaluation rather than extrapolation from findings obtained with the PPI class as a whole.

However, gastric acid secretion by parietal cells is controlled by a complex interplay of several different pathways that ultimately lead to the activation of the H^+^/K^+^-ATPase by various second messengers. Thus, whereas an increase in gastric acidity can be achieved by stimulation of the secretory pathways, a decrease in gastric acidity is primarily achieved through inhibition of these pathways. Pantoprazole covalently modifies the H^+^/K^+^-ATPase in the gastric parietal cells, resulting in long-lasting specific inhibition of gastric acid secretion [3,4,5]. Of note, the time required to restore the H^+^/K^+^-ATPase secretory capacity in parietal cells, i.e., the de novo synthesis of new H^+^/K^+^-ATPase molecules, greatly exceeds the plasma half-life of the parent drug. Furthermore, because of differences in tissue versus plasma exposure, intracellular versus extracellular localization, and the duration of interaction with the target versus with other (tissue) proteins, the non-gastric actions of PPIs may differ considerably from their well-established acid-inhibitory effects in the stomach.

Pantoprazole and other PPIs have been studied extensively for gastrointestinal diseases, and emerging research has investigated whether their pharmacology may also influence proton-dependent processes outside the gastrointestinal tract. However, the biological effects of one PPI cannot automatically be attributed to the entire class, nor can findings obtained with other PPIs be assumed to apply directly to pantoprazole. Proton-dependent processes are not limited to the gastrointestinal tract. Such processes are integral to many other physiological functions, including cellular homeostasis, organelle acidification, lysosomal degradation, vesicular transport, transmembrane transport, and mitochondrial function. The extracellular microenvironment of cells is also influenced by pH. Due to this unique set of features, drugs that interfere with proton handling may exert pharmacological effects that extend far beyond their primary site of action in the gastrointestinal tract. In fact, many of the PPIs have been considered as candidates for the use of drugs as “repurposing” agents in the treatment of a variety of human cancers and of other non-gastrointestinal diseases associated with altered stress responses [7,8,9,10]. The reported effects should be interpreted according to the specific compound, experimental model, and level of mechanistic evidence. Some studies suggest pantoprazole-specific molecular effects, whereas others report changes in signaling pathways, cellular stress responses, apoptosis, or treatment sensitivity that may arise downstream of a more proximal perturbation. Changes in pathway activity or cellular phenotype alone do not establish direct binding to, or inhibition of, the corresponding molecular target. A direct mechanism requires appropriate evidence of target engagement and, where feasible, functional experiments demonstrating that the proposed target contributes causally to the observed effect. Conversely, changes in oxidative stress, inflammatory mediators, autophagy, or treatment sensitivity may reflect indirect consequences of altered cellular homeostasis rather than independent primary actions of pantoprazole. Findings obtained with other PPIs or chemically distinct compounds should not be treated as direct evidence of pantoprazole activity. Furthermore, experimental pathway modulation does not by itself establish therapeutic efficacy in humans.

The term “drug pleiotropy” in modern pharmacology refers to the unusual capacity of most drugs to act on several different targets and pathways rather than on a single molecular receptor. A single drug typically acts on a primary target and then may subsequently influence additional downstream pathways through various pathways. Thus, a drug’s effects on different pathways are not necessarily attributable to direct interactions with those targets but may instead result from modulation of downstream signaling by the primary target. Accordingly, when studying a drug’s pleiotropism, it is important to distinguish between its interactions with a major target and its subsequent effects on other downstream targets. In this study, we have reported the diverse ways in which the different effects of pantoprazole could be considered as part of its pleiotropic pharmacology and what is currently known about its various non-gastric actions.

Cancer represents the major disease class in which the effects of pantoprazole have been most extensively investigated. Cancer cells differ from most normal cells in their metabolic requirements and in their regulation of intracellular as well as extracellular pH. Their high rate of glycolysis, for instance, results in the production of large amounts of acid, which malignant cells counteract through increased activity of proton transporters to maintain a near-alkaline intracellular environment. The acidic tumor microenvironment can, in turn, support several characteristics of malignancy, including invasive growth, angiogenesis, metabolic shift into tumor-specific marker-related pathways, and resistance to therapy [11,12,13]. Proton transport and organelle acidification therefore represent potentially exploitable vulnerabilities. Experimental studies have demonstrated that pantoprazole can interfere with several processes associated with this phenotype, including autophagy, lysosomal function, apoptosis, survival signaling, and drug transport [7,11,14]. These findings have subsequently been extended across multiple tumor types, generating a broad but heterogeneous body of literature on the potential anticancer activity of pantoprazole.

A second major domain of investigation concerns inflammation and oxidative stress. These processes are intimately interconnected because Reactive oxygen species (ROS) can activate inflammatory transcriptional pathways, while inflammatory cytokines and immune-cell activation can generate additional oxidative stress [15,16,17,18]. Experimental studies have reported that pantoprazole attenuates inflammatory signaling and oxidative injury in models of myocardial and renal ischemia–reperfusion, cisplatin nephrotoxicity, cyclophosphamide-induced cystitis, and sepsis-associated organ injury [19,20,21,22,23,24]. Although these findings suggest a potentially coherent stress-modulating phenotype, they should not automatically be interpreted as evidence that pantoprazole functions as a conventional antioxidant or anti-inflammatory drug. The observed reductions in oxidative markers may instead reflect modulation of upstream signaling pathways or tissue injury.

The role of PPIs in *Helicobacter pylori* eradication is well established and is largely attributable to gastric acid suppression, which improves the activity and stability of acid-sensitive antibiotics [25,26,27]. More recent experimental work has suggested that pantoprazole and structurally related compounds may additionally interfere with bacterial resistance mechanisms, including metallo-β-lactamases, proton-dependent stress adaptation, and biofilm-associated survival [9,10,28,29,30,31,32,33,34,35]. Of particular interest is the potential therapeutic value of a resistance-modifying agent that augments the activity of existing antibiotics rather than serving as an alternative treatment for every clinical situation.

In addition to cancer, inflammation, and infection, the literature increasingly suggests that pantoprazole may affect glucose homeostasis, neurodegenerative diseases, hepatic fibrosis, and iron metabolism [36,37,38,39,40,41,42,43]. However, the evidence supporting these proposed ‘new indications’ differs substantially in study design and experimental setting and therefore should not be considered equivalent in terms of therapeutic potential. For instance, some studies were performed in cell cultures, others in animal models, human observational studies, and a few even in clinical randomized trials; these different levels of evidence should be interpreted accordingly.

The rationale for investigating pantoprazole beyond its established indications rests on its clinical familiarity, characterized pharmacology, comparatively favorable interaction profile relative to certain alternative PPIs, and emerging experimental observations. Importantly, this rationale reflects the feasibility of investigating pantoprazole as a repurposing candidate, rather than evidence that it is superior to other PPIs or that its proposed non-gastric effects are established clinical benefits. These considerations provide a basis for research but do not establish efficacy for a new indication. A critical issue is whether the concentrations and exposure durations associated with experimental effects can be achieved in the relevant human tissue. This question is particularly important when continuous high-micromolar exposure in cultured cells is compared with the transient systemic exposure following conventional oral dosing. Accordingly, this review distinguishes established pharmacological effects from mechanistic hypotheses, preclinical findings, and clinical evidence, while evaluating the extent to which each proposed effect is supported by exposure-relevant and disease-specific data. This review aims to move beyond a descriptive list of the reported effects of pantoprazole and provide a more integrated view of the emerging pharmacology of this drug by highlighting the potential interrelationships among effects related to proton and pH homeostasis, organelle function, oxidative stress, inflammation, autophagy and cellular survival across different diseases. The exposure–mechanism gap, the distinction between class effects and pantoprazole-specific effects, the limitations of predominantly preclinical evidence, and the lessons that can be derived from the few human studies are important issues addressed in this review. The objective of this study is to provide a robust scientific basis for the concept of pantoprazole repurposing rather than merely cataloging pharmacological observations.

## 2. Literature Search Strategy and Evidence Framework

We conducted a narrative literature search to identify publications addressing the established pharmacology of pantoprazole and its proposed systemic biological effects. The databases searched were PubMed, Scopus, Web of Science, and Google Scholar. The search covered publications available from January 2005 to August 2026. Search terms included “pantoprazole,” “proton pump inhibitor,” “pleiotropic effects,” “drug repurposing,” “proton transport,” “intracellular pH,” “V-ATPase,” “autophagy,” “oxidative stress,” “inflammation,” “cancer,” “antimicrobial resistance,” “biofilm,” “glycemic control,” “neuroprotection,” “liver fibrosis,” and “thalassemia.” These terms were combined with relevant disease-specific and mechanistic keywords using appropriate Boolean operators. Reference lists of relevant publications were also examined to identify additional eligible studies. Recent publications were prioritized to capture emerging findings, while older studies were retained when they provided important evidence concerning pantoprazole’s established pharmacology or foundational mechanisms.

Eligible publications included original in vitro, biochemical, animal, observational, and clinical studies that investigated pantoprazole’s pharmacological properties, proposed systemic mechanisms, disease-related effects, safety, or clinical outcomes. Relevant review articles were included to provide context, identify foundational studies, and support interpretation of the evidence. Publications involving other proton pump inhibitors (PPIs) or chemically distinct compounds were considered only when they provided relevant contextual information; their findings were not treated as direct evidence of a pantoprazole-specific effect. Publications were excluded when they were unrelated to the review objectives, duplicate records, or reports that did not provide sufficient information to evaluate their relevance. Where a publication concerned a PPI class effect rather than pantoprazole specifically, this distinction was retained during evidence interpretation. As this was a narrative review rather than a systematic review, the literature selection was guided by relevance to the review objectives, mechanistic significance, study design, and translational applicability rather than by a prespecified systematic screening protocol or quantitative study-selection target. Accordingly, the search was intended to support a critical narrative synthesis and was not designed to generate a comprehensive numerical accounting of all records identified, screened, excluded, and included. To facilitate critical comparison of the available evidence, Table 1 summarizes representative studies according to their experimental models, principal findings, and translational relevance. The studies vary substantially in design, biological context, and outcome assessment; therefore, their findings should be interpreted within the limitations of the respective experimental systems. In particular, activity observed in cultured cells or animal models does not necessarily indicate that comparable effects occur in humans at clinically achievable exposure. Differences in exposure conditions, pharmacokinetics, tissue distribution, and biological context may influence the reproducibility and clinical relevance of the reported effects. Accordingly, the evidence is classified according to its experimental or clinical setting, and mechanistic observations are distinguished from preclinical efficacy and demonstrated clinical benefit.

As shown in Table 1, the evidence spans in vitro experiments, animal models, and human studies, with substantial differences in exposure conditions and translational maturity. Findings obtained through cellular pathway modulation or animal disease models are therefore interpreted as mechanistic or preclinical evidence, rather than as proof of clinical effectiveness. Where experimental concentrations or doses exceed conventional human exposure, or where the necessary pharmacokinetic information is unavailable, this limitation is considered when interpreting the proposed therapeutic relevance.

## 3. From Proton Pump Inhibition to Systemic Pleiotropy

Proton homeostasis is fundamental to intracellular physiology and extends far beyond gastric acid secretion by parietal cells. The proton gradient is a critical parameter for the proper functioning of intracellular organelles, membrane-bound transporters, and vesicular transport systems and plays a critical role in the maintenance of appropriate intracellular pH [50]. Proteolytic breakdown of proteins by lysosomes requires an acidic environment, whereas the endosomal and secretory pathways require tightly controlled proton gradients for the proper maturation of transport and secretory vesicles [51]. Mitochondrial function is also linked to proton gradients, although through distinct membrane and bioenergetic mechanisms. Consequently, perturbation of proton distribution can potentially influence multiple cellular processes simultaneously [52].

Many of these processes are severely perturbed in diseased cells, which may exhibit unique metabolic adaptations to support high proliferation rates. For example, many tumors produce large amounts of acid during their metabolism, which must be extruded from the cell to maintain an appropriate intracellular pH. Other pathogenic organisms can encounter highly variable environments during their life cycle, and in many cases, maintaining an appropriate intracellular pH is often crucial for their survival. Cells in inflammatory or ischemic tissues may contain damaged mitochondria that generate high amounts of Reactive Oxygen Species (ROS), while many membrane transport processes may also be severely altered, resulting in cellular ion imbalances that can exacerbate tissue damage. As a result, a drug that can modulate these processes across different tissues could produce vastly different clinical effects.

The pathways reported to be affected by pantoprazole encompass several interconnected cellular systems rather than necessarily representing multiple direct molecular targets. Autophagy, apoptosis, nuclear factor kappa B (NF-κB), mitogen-activated protein kinase (MAPK), signal transducer and activator of transcription 3 (STAT3), phosphoinositide 3-kinase/protein kinase B/mechanistic target of rapamycin (PI3K/Akt/mTOR), Wnt/β-catenin signaling, and drug-efflux transporters have all been implicated in cellular adaptation to stress [53,54,55,56]. However, changes in these pathways do not, by themselves, demonstrate that pantoprazole directly binds to or inhibits their constituent proteins. Such changes may occur downstream of altered proton handling, lysosomal function, cellular stress, or other upstream perturbations. The functional consequences are also likely to vary by cell type and disease context: a stress response may contribute to tumour-cell death, protection against ischemic tissue injury, or altered microbial susceptibility. Therefore, each proposed mechanism should be classified according to the level of evidence available, distinguishing direct target engagement from pathway modulation and downstream phenotypic association. Accordingly, gastric H+/K+-ATPase inhibition represents the established primary pharmacological action of pantoprazole, whereas the reported effects on STAT3, NF-κB, MAPK, PI3K/Akt/mTOR, Wnt/β-catenin, autophagy, and apoptosis should generally be interpreted as pathway modulation or downstream responses unless direct target engagement and causal involvement have been demonstrated in the relevant experimental system.

The translational relevance of the proposed effects on intracellular proton homeostasis depends on whether pantoprazole or its active species can reach the relevant extragastric tissues at sufficient concentrations and for an adequate duration. Following a conventional oral dose of 40 mg, pantoprazole reaches a reported peak serum concentration of approximately 2.5 µg/mL (about 6.5 µM), with a terminal elimination half-life of approximately one hour [57,58]. These pharmacokinetic characteristics should be considered when interpreting experimental studies involving prolonged exposure to high-micromolar concentrations. For example, pantoprazole was tested at 50 µM for 72 h in colorectal cancer cell models, a nominal concentration approximately 7.7 times the total plasma peak concentration associated with a 40 mg oral dose [45]. This comparison is approximate: total plasma concentration, unbound concentration, culture-medium exposure, and tissue exposure are not interchangeable. At present, the available evidence does not establish that routine oral pantoprazole dosing reproducibly alters intracellular pH in human extragastric tissues. Exposure-matched experiments with direct measurements of intracellular pH, drug accumulation, activation kinetics, and target engagement are therefore needed. Experimental studies have directly examined the effects of pantoprazole on intracellular pH in cancer-cell models. In gastric adenocarcinoma cells, significant intracellular-pH changes were reported at pantoprazole concentrations of 10–100 µg/mL, whereas no significant change was reported at 1 µg/mL [59]. In prostate, breast, and ovarian cancer-cell models, pantoprazole at 100 µM was reported to modify intracellular pH in a cell- and extracellular-pH-dependent manner, although a clear response was not observed in every cell line [48]. In pancreatic ductal adenocarcinoma cells, pantoprazole also inhibited cellular H+ extrusion and increased extracellular pH; however, these experiments used concentrations of 50–100 µM, rather than concentrations representative of standard oral exposure [12]. Collectively, these studies demonstrate that pantoprazole can affect cellular pH regulation under experimental conditions, but they do not establish that comparable effects occur reproducibly in human extragastric tissues at standard therapeutic exposure.

Pantoprazole’s proposed pleiotropy is therefore best evaluated as context-dependent modulation of interacting cellular systems rather than as evidence of nonspecific direct interaction with numerous unrelated molecular targets. A downstream pathway change is insufficient, on its own, to establish a direct mechanism or clinically meaningful activity. Mechanistic validation should identify the earliest reproducible molecular event and test causality using orthogonal approaches, including target-engagement assays, pharmacological controls, genetic perturbation, and rescue experiments where appropriate. These experiments should also be conducted at concentrations relevant to the intended clinical application. Only after the primary perturbation, its downstream consequences, and its relationship to a disease-relevant phenotype have been established should the mechanism be used to justify further therapeutic development.

Acid-dependent activation is an important consideration when interpreting proposed extragastric effects of pantoprazole. Pantoprazole is an acid-activated prodrug whose conversion to reactive intermediates is favoured in the highly acidic secretory canaliculi of gastric parietal cells. By comparison, the extracellular pH of many solid tumours is approximately 6.5–7.0, whereas lysosomal luminal pH is generally around 4.5–5.0 [11,60,61,62,63]. These differences raise an important mechanistic question: whether the parent drug is activated sufficiently rapidly in extragastric compartments to produce the proposed biological effects. An acidic tumour microenvironment or lysosomal lumen does not, by itself, establish adequate formation of active pantoprazole species, cellular accumulation, or target engagement. The relatively high acid stability of pantoprazole may be advantageous in some pharmacological respects, but it could also limit activation-dependent effects outside the highly acidic gastric secretory compartment. The proposed extragastric mechanisms should therefore be evaluated by measuring local pH, activation kinetics, parent-drug and active-species concentrations, and target engagement. Until these measurements are available, acid-dependent activation outside the stomach should be considered a testable hypothesis rather than an established explanation for all reported biological effects.

## 4. Cancer and Therapeutic Resistance

Much of the experimental literature investigating pantoprazole’s proposed effects beyond gastric acid suppression concerns cancer biology and therapeutic resistance. Initially, it seemed logical that because malignant cells have an abnormal pH-type that maintains an alkaline intracellular environment, even while producing large amounts of acidic metabolites, interference with their proton-transporting systems could be particularly effective. Many malignant cells not only produce acidic metabolites but also create an acidic extracellular environment by means of ion-regulatory systems, which in turn promotes tumor invasion, angiogenesis and resistance to cancer therapy. Inhibiting such a system is therefore expected to have far more effects on malignant cells than inhibiting any single component of such a system. The anticancer evidence should nevertheless be interpreted according to the tumour model, experimental concentration, exposure duration, and mechanistic validation available for each study. Tumour-cell cytotoxicity, chemosensitization, and modification of the tumour microenvironment are distinct proposed roles and should not be treated as equivalent. An effect on cell viability does not establish a clinically useful anticancer treatment, and an enhanced response to a combination does not establish pharmacological synergy unless a suitable interaction analysis has been performed. Based on the currently available evidence, pantoprazole should not be classified primarily as a direct cytotoxic anticancer agent. Rather, its most plausible investigational role is as a potential chemosensitizing agent, with additional context-dependent effects on cellular stress responses and the tumour microenvironment. However, this interpretation remains provisional because the relative contribution of each mechanism varies across experimental models, and clinically meaningful chemosensitization or microenvironment modulation has not been established consistently in patients.

Experimental evidence suggests that pantoprazole can alter autophagy-associated processes in selected cancer models [7]. Autophagy is a homeostatic process by which cells generally maintain themselves by degradation and recycling of damaged proteins and organelles. However, malignant cells also utilize autophagy to sustain their growth in nutrient-deprived, hypoxic tumors as well as during cancer therapy. By inhibiting protein degradation and blocking key steps in autophagic processing, for example, by inhibiting the LC3-converting proteases, the amount of damaged cellular material that can be amassed by tumor cells is increased, thereby leading to impaired cancer cell survival during stress. Such a concept is very attractive for a repurposing approach, since many established drugs lack sufficient direct anticancer activity but can be used to increase the effectiveness of already established cancer therapeutics by targeting specific resistance mechanisms.

Pantoprazole has been investigated for its effects on gastric cancer cells in several experimental systems, including studies examining cell proliferation, apoptosis, and specific cancer-associated signaling pathways [64,65,66,67]. These studies suggest that pantoprazole can inhibit cancer-cell proliferation and promote apoptosis under the experimental conditions investigated. Cancer stem cells contribute to cancer initiation, recurrence, metastasis and resistance to anticancer drugs. In a series of experiments, Wnt/β-catenin signaling was downregulated and gastric cancer stem-cell markers (Cluster of differentiation 44 (CD44), Cluster of differentiation 24 (CD24), ATP-binding cassette subfamily G member 2 (ABCG2), Epithelial cell adhesion molecule (EpCAM), and Leucine-rich repeat-containing G-protein-coupled receptor 5 (Lgr5) were decreased by pantoprazole in gastric cancer cells. Pantoprazole also reduced sphere formation under stem-cell-like conditions, and treated cancer cells were sensitized to 5-fluorouracil [64,65]. Moreover, pantoprazole inhibited endothelial tube formation by activated hepatic stellate cells and affected EMT (epithelial to mesenchymal transition) marker expression [66]. In addition, the expression of telomerase reverse transcriptase was downregulated by pantoprazole, and this was accompanied by reduced metastasis in experimental models [67]. In gastric cancer models, pantoprazole-associated induction of Src homology region 2 domain-containing phosphatase-1 (SHP-1) and subsequent downregulation of Janus kinase 2 (JAK2)/signal transducer and activator of transcription 3 (STAT3) signaling have also been implicated in its effects on cellular behavior [64]. STAT3 regulates genes involved in cell survival, proliferation, and inflammation; however, the extent to which its modulation contributes to pantoprazole-associated effects requires interpretation in the context of the specific experimental model. Related but distinct evidence comes from a mouse model of cancer cachexia established using C26 colon adenocarcinoma cells. In this model, oral pantoprazole treatment attenuated body-weight loss and skeletal muscle wasting in a dose-dependent manner, alongside reductions in circulating interleukin-6 and tumour necrosis factor-α, decreased JAK2/STAT3 expression and phosphorylation in skeletal muscle, and reduced expression of the muscle protein-degradation-associated factors MuRF1 and Fbx32 [68]. These findings suggest that pantoprazole-associated modulation of JAK2/STAT3 signaling may also occur in the context of cancer-associated systemic inflammation and muscle wasting. Nevertheless, the cachexia study does not establish direct binding or direct inhibition of STAT3 by pantoprazole, nor does it demonstrate clinical effectiveness in patients. More broadly, the relative contributions of direct molecular interactions, upstream cellular stress, and downstream pathway modulation remain to be distinguished. The reported effects should also be interpreted in relation to the concentrations and exposure durations used, because reproducible activity in cultured cells or animal models does not establish that the same effects occur in human tumours at clinically achievable exposure.

Pantoprazole has been demonstrated to inhibit TOPK (T-cell-originated protein kinase), a serine/threonine kinase involved in mitotic processes and tumor-cell proliferation, in colorectal cancer cell lines [44]. Pantoprazole has also been reported to interfere with histone H3 phosphorylation mediated by TOPK in tumor cells and to suppress experimental colorectal tumor growth [44]. Enhanced anticancer activity in combination with 5-fluorouracil has also been reported under acidic tumor-microenvironment conditions [45]. A clinical study investigating proton pump inhibitor (PPI) use in patients with advanced colorectal cancer reported an association with increased chemosensitivity [45]. However, this retrospective clinical association does not establish that pantoprazole itself improves clinical outcomes. Further studies with appropriate clinical endpoints are required to determine whether these experimental findings translate into clinical benefit.

Pancreatic ductal adenocarcinoma (PDA) has a complex tumor microenvironment characterized by hypoxia, metabolic stress, extensive stromal involvement, and altered extracellular acidity [12,13,69]. These features provide a rationale for investigating whether interventions affecting proton handling could influence tumor-cell behavior and tumor–stroma interactions. The potential for proton-handling interventions to modify the pancreatic tumor microenvironment remains a hypothesis requiring direct experimental and translational evaluation. In gastric cancer cell models, pantoprazole suppressed cell proliferation and enhanced chemosensitivity, with inhibition of IL-6/STAT3 signaling implicated in these effects [70]. These findings provide a rationale for further investigation but should not be interpreted as evidence of demonstrated therapeutic efficacy in pancreatic cancer or as proof that the proposed mechanisms operate at clinically achievable pantoprazole exposures.

The effect of pantoprazole in a model of experimental hepatic carcinogenesis was also evaluated. Pretreatment with pantoprazole significantly reduced the number and size of precancerous lesions and improved the tissue architecture [46]. Most importantly, the study found that pantoprazole treatment significantly affected the process of extracellular-vesicle (EV) biology. In particular, the authors found reduced expression of several genes associated with exosome secretion following pantoprazole treatment. Moreover, they showed that the drug affected the release of regulatory molecules involved in the regulation of EVs release [46]. Because EVs contribute to intercellular communication, transfer of regulatory RNA and tumor progression, modulation of this pathway provides a mechanism through which pantoprazole could influence the tumor microenvironment beyond direct effects on malignant-cell proliferation.

Drug resistance represents a recurring theme across the anticancer literature. In vincristine-resistant oral epidermoid carcinoma models, pantoprazole increased intracellular accumulation of vincristine by suppressing P-glycoprotein-mediated drug efflux [47]. The drug may also modify lysosomal sequestration because acidic intracellular compartments can trap certain therapeutic molecules and reduce their availability at cytosolic or nuclear targets. Pantoprazole-associated alteration of lysosomal acidity has therefore been proposed as another mechanism through which intracellular drug distribution may be modified [47]. Similar effects on P-glycoprotein and multidrug resistance-associated protein 1 have been observed in leukemic models, accompanied by modulation of PI3K/Akt/mTOR signaling and increased apoptosis [71].

The evidence from glioma, breast and cervical cancer extends the mechanistic framework. In glioma models, pantoprazole induces mitochondrial apoptosis, increases oxidative stress and suppresses NF-κB-associated inflammatory signaling [72,73]. In breast cancer models, pantoprazole has been identified as a potential inhibitor of Forkhead box M1 (FOXM1), a transcriptional regulator associated with cell-cycle progression and malignant proliferation [74]. In cervical cancer, pantoprazole increases cisplatin sensitivity through suppression of cisplatin-induced autophagy, with experimental reversal by rapamycin supporting the importance of autophagic signaling [75]. All these investigations show that the claimed anticancer effects are not specific to one tissue or molecular mechanism.

Pantoprazole in combination with vitamin C has been investigated in metastatic castration-resistant prostate cancer models [48]. The reported effects on metabolic activity and xenograft tumour growth provide preclinical evidence for the combination under the conditions tested but do not establish clinical efficacy. In the PANDORA phase II study, high-dose intravenous pantoprazole was administered with docetaxel and prednisone in men with metastatic castration-resistant prostate cancer [49]. The study did not meet its prespecified criterion for proceeding to the second stage. This result highlights the translational gap between preclinical activity and clinical benefit and reinforces the need for exposure characterization, target-engagement evidence, and prospectively defined clinical endpoints.

A further limitation in translating the reported anticancer effects concerns the relationship between experimental concentrations and exposures achievable in clinical practice. Following a single oral dose of pantoprazole 40 mg, the reported maximum plasma concentration is approximately 2.5 mg/L, equivalent to about 6.5 µM total pantoprazole [57,58]. By comparison, Koh et al. investigated pantoprazole concentrations of 40–160 µg/mL (approximately 104–418 µM) in gastric cancer models examining SHP-1/STAT3 signaling [64], while Huang et al. reported enhanced cisplatin sensitivity following pantoprazole pretreatment at 20 µg/mL (approximately 52 µM) [70]. These nominal experimental concentrations substantially exceed the total plasma peak concentration reported after standard oral dosing. Pantoprazole has also been investigated at micromolar concentrations in gastric cancer stem-like cell models, including studies of Wnt/β-catenin signaling, metastasis, and angiogenesis [65,66]. However, concentration comparisons must be interpreted cautiously because nominal cell-culture concentrations, total plasma concentrations, unbound drug concentrations, and tumour-tissue exposure are not interchangeable. Differences in protein binding, exposure duration, administration route, and tissue distribution may influence the relationship between in vitro activity and clinical exposure. Although high-dose intravenous pantoprazole has been investigated in the PANDORA phase II trial [49], clinical administration alone does not demonstrate that the concentrations required for the reported experimental effects were achieved in tumour tissue. Accordingly, the translational relevance of these findings remains uncertain unless supported by pharmacokinetic and exposure–response data, together with evidence of target engagement at clinically relevant exposures.

When viewed together, the anticancer literature suggests that pantoprazole may influence several interconnected mechanisms involved in tumour-cell survival and therapeutic resistance, including proton and pH regulation, autophagy, apoptosis, survival signaling, drug transport, lysosomal sequestration, cancer stem-cell phenotypes, angiogenesis, epithelial–mesenchymal transition, and extracellular-vesicle communication [11,14,76]. Nevertheless, the strength of evidence differs substantially across tumour types, experimental models, and proposed mechanisms. The concentration-dependent effects observed in cell cultures should be distinguished from effects demonstrated at clinically achievable exposures, and evidence of reduced cell viability does not establish clinically useful cytotoxic activity. Similarly, increased sensitivity to chemotherapy does not establish pharmacological synergy without appropriate interaction analysis, while changes in tumour acidity or cellular stress markers do not establish clinically meaningful tumour-microenvironment modulation without direct measurements. The available evidence therefore supports further investigation of pantoprazole as a potential chemosensitizing agent rather than establishing it as an effective anticancer treatment. Determining its translational potential will require pharmacokinetic and exposure–response studies, evidence of target engagement at clinically relevant exposures, and prospective clinical trials demonstrating meaningful improvements in patient outcomes.

## 5. Anti-Inflammatory, Antioxidant and Organ-Protective Effects

Inflammation and oxidative stress are part of numerous diseases and recently have been characterized as dynamic systems of tissue damage rather than as two independent harmful situations. ROS can trigger inflammatory transcription factors like NF-κB whereas inflammatory cytokines can increase oxidative stress by inducing mitochondrial damage and by activating enzymes that produce ROS [15,16,17,18]. The resulting feedback loop can amplify cellular damage and contribute to apoptosis, endothelial dysfunction and organ failure.

Renal ischemia–reperfusion injury provides an important model because restoration of blood flow produces rapid ROS generation, inflammatory activation and mitochondrial dysfunction. Pantoprazole has been shown to attenuate activation of MAPK and NF-κB pathways and reduce inflammatory mediators including Tumor necrosis factor-alpha (TNF-α), Interleukin-1 beta (IL-1β) and Interleukin-6 (IL-6) [20]. Changes in the Bcl-2-associated X protein (Bax)/Bcl-2 balance and reductions in cleaved caspase-3 suggest that the drug also suppresses downstream apoptosis. Complementary studies demonstrate improvements in antioxidant status and reductions in lipid peroxidation following pantoprazole administration [21]. Similar findings have been reported in myocardial ischemia–reperfusion models, in which pantoprazole reduced inflammatory and oxidative injury while improving indices of endothelial and nitric-oxide-related function [19]. The consistency of these observations across two major organs suggests that the effects may reflect a broader stress-modulating phenotype rather than a tissue-specific phenomenon.

A randomized clinical study in patients receiving cisplatin for head and neck cancer reported a renal-protective effect associated with pantoprazole administration [77]. This finding provides human evidence that warrants further evaluation, although its clinical significance, reproducibility, and implications for cancer-treatment efficacy require careful assessment [77]. Cisplatin-induced nephrotoxicity is associated with ROS production, mitochondrial dysfunction, inflammation and apoptosis. Pantoprazole has been found to increase antioxidant defense, glutathione levels, and decrease inflammatory cytokines and apoptotic signaling in experiments [22].

It has also been reported that the protective effect of pantoprazole against cyclophosphamide-induced hemorrhagic cystitis was associated with reduced lipid peroxidation, decreased inflammatory mediator levels, and improved glutathione levels [23]. More recently, pantoprazole was also shown to exert protective effects in experimental sepsis models. The drug decreased the production of systemic inflammatory mediators and attenuated lung and kidney injury, with the Ras homolog family member A/Rho-associated coiled-coil-containing protein kinase (RhoA/ROCK) pathway implicated in the underlying molecular mechanism [24]. Although NF-κB and MAPK pathways have been primarily implicated in these actions of pantoprazole, other signaling pathways may also contribute.

A large nationwide cohort study found that PPI use was associated with lower mortality and fewer severe cases among patients with mild acute pancreatitis [78]. Because this was a retrospective study, the association cannot establish causality and may be affected by confounding, differences in patient characteristics, or indication-related factors. Furthermore, evidence concerning PPI use as a class should not automatically be interpreted as evidence of a pantoprazole-specific effect. The reported association may provide a rationale for further investigation, but prospective studies are needed to determine whether pantoprazole itself influences clinically relevant outcomes in acute pancreatitis.

An important consideration in translating these findings is whether the protective effects observed in experimental models occur at pantoprazole exposures achievable in clinical practice. Although animal studies report improvements in inflammatory, oxidative-stress, and apoptotic markers, these findings alone do not establish that comparable effects occur at standard therapeutic doses in humans. Such an assessment requires comparison of the doses and systemic exposures used in preclinical studies with human pharmacokinetic data, together with consideration of the concentrations required to produce the reported biological effects. Differences in species, routes of administration, treatment duration, and disease models may further influence the translational relevance of the findings. Accordingly, the clinical applicability of pantoprazole-mediated organ protection remains uncertain where exposure–response relationships have not been established, and future studies should determine whether protective effects can be reproduced at clinically relevant exposures.

**Figure 1:**
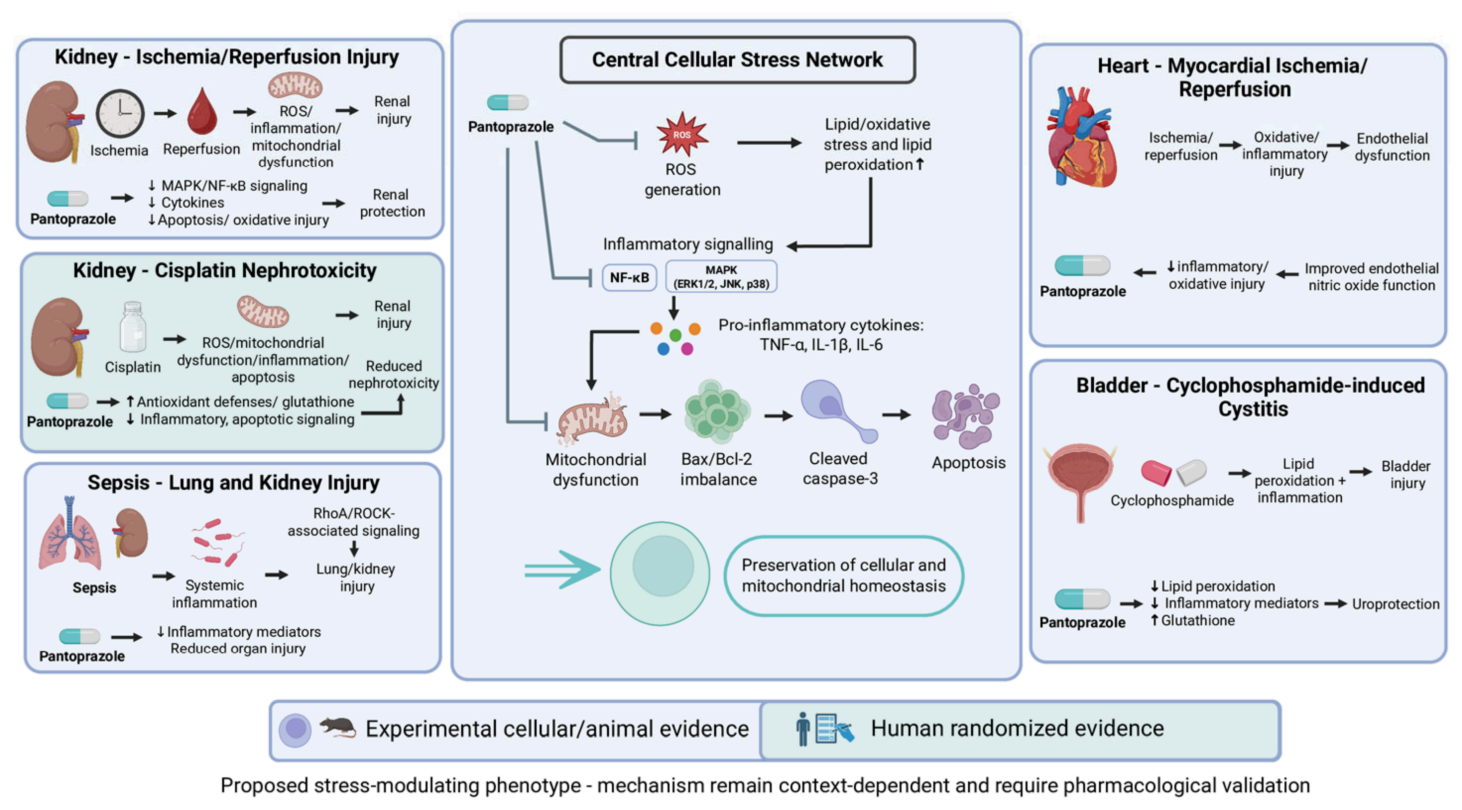
Central cellular stress-modulating effects of pantoprazole. The diagram illustrates the protective effects of pantoprazole against diverse forms of tissue injury through modulation of interconnected oxidative, inflammatory, mitochondrial, and apoptotic pathways. Created in BioRender. Satyam, D.S.M. (2026) https://BioRender.com/l5ahbr0. Abbreviations: Bax, Bcl-2-associated X protein; Bcl-2, B-cell lymphoma 2; ERK1/2, extracellular signal-regulated kinases 1 and 2; IL-1β, interleukin-1 beta; IL-6, interleukin-6; JNK, c-Jun N-terminal kinase; MAPK, mitogen-activated protein kinase; NF-κB, nuclear factor kappa B; ROS, reactive oxygen species; RhoA/ROCK, Ras homolog family member A/Rho-associated coiled-coil-containing protein kinase; TNF-α, tumor necrosis factor-alpha.

Overall, the available evidence suggests that pantoprazole may influence inflammatory, oxidative-stress, and apoptotic pathways in selected experimental models. However, the observed reductions in oxidative-stress markers may be secondary to changes in upstream inflammatory signaling, mitochondrial function, or the severity of tissue injury rather than evidence of direct antioxidant activity. As illustrated in Figure 1, the proposed effects are best interpreted as modulation of interconnected cellular stress responses. Future studies should distinguish direct chemical antioxidant effects from indirect biological responses, establish the earliest causally relevant molecular events, and determine whether these effects occur at pharmacologically relevant concentrations.

## 6. Emerging Extra-Gastrointestinal Effects of Pantoprazole

### 6.1. Antimicrobial Activity and Antimicrobial Resistance

Pantoprazole’s role in *Helicobacter pylori* eradication is primarily related to gastric acid suppression within combination treatment regimens, which can improve the activity and stability of acid-sensitive antibiotics [25,26,27]. This established contribution should be distinguished from direct antimicrobial effects attributed to pantoprazole itself. Experimental studies have investigated pantoprazole, related compounds, and PPIs as potential modifiers of bacterial enzymes, metallo-β-lactamases, antibiotic susceptibility, and biofilm-associated processes [9,10,28,29,30,31,32,33,34,35]. However, studies of pantoprazole derivatives should not be treated as direct evidence for the parent drug unless pantoprazole itself is tested.

The potential inhibition of bacterial resistance determinants and enhancement of meropenem activity are promising experimental observations, but their translational relevance depends on the compound tested, bacterial species, assay conditions, concentration, and method used to assess synergy [9,28,29,30,31]. Similarly, findings involving Enterococcus faecalis and oral biofilms should be interpreted according to the specific PPI or compound examined [32,33,34,35,79]. In vitro inhibition of bacterial enzymes does not establish that the necessary concentration can be achieved at a human infection site or that treatment improves microbiological eradication or patient outcomes.

The antimicrobial potential of pantoprazole should therefore be evaluated separately from its established role in acid suppression. Further work should determine whether parent pantoprazole produces reproducible antimicrobial or resistance-modifying effects at relevant exposures, whether these effects are additive or synergistic with antibiotics, and whether they can be achieved safely in vivo. Potential effects on commensal organisms should also be considered, because long-term PPI use has been associated with changes in gastrointestinal microbial composition and other gastrointestinal outcomes [80]. At present, direct antimicrobial repurposing should be considered experimental rather than established clinical practice.

### 6.2. Metabolic Effects and Glucose Regulation

The possible metabolic effects of PPIs may involve several mechanisms, including changes in circulating gastrin during sustained acid suppression, pancreatic β-cell function, gastrointestinal microbial composition, and nutrient handling. In the Goto–Kakizaki diabetic rat, chronic pantoprazole administration increased gastrin levels in the circulation, stomach, and pancreas and was associated with changes in glucose metabolism, β-cell mass, and β-cell function [36]. These findings support further investigation of a possible relationship between pantoprazole, gastrin signaling, and glucose regulation, but they do not independently establish a clinically meaningful glucose-lowering effect in humans.

Some clinical studies have reported changes in fasting or postprandial glucose levels among diabetic patients receiving pantoprazole for short periods [40,81,82]. Systematic reviews and meta-analyses of randomized clinical trials have also reported modest evidence of beneficial effects of PPIs on glycemic control, particularly in type 2 diabetes, during short treatment periods [39,40]. These results should be interpreted in relation to study duration, sample size, concomitant antidiabetic treatment, baseline metabolic status, and the magnitude of the observed change. Class-level evidence should not automatically be attributed to pantoprazole alone.

Further research should determine whether the reported effects are reproducible, whether gastrin or β-cell changes mediate the observed response, and whether any effect is sustained and clinically meaningful. Changes in glucose-related biomarkers should not be equated with demonstrated improvement in long-term diabetes outcomes. Pantoprazole should therefore be regarded as an investigational modifier of glucose-related pathways rather than an established glucose-lowering therapy.

### 6.3. Neurological Effects

Experimental studies have investigated the potential neurological effects of pantoprazole in models involving seizure-associated responses, oxidative stress, and inflammation. In a pentylenetetrazole-induced seizure model using SH-SY5Y neuronal-like cells, pantoprazole treatment was associated with reductions in several proinflammatory and oxidative-stress mediators and apparent protection of neuronal architecture [41]. These observations are preliminary and do not establish clinical neuroprotection or antiseizure efficacy. Changes in inflammatory or oxidative markers may reflect secondary responses to altered cellular stress rather than direct interaction with a neuronal target.

Further investigation should establish whether pantoprazole or relevant metabolites reach the central nervous system at sufficient concentrations, demonstrate target engagement, and produce reproducible functional benefits in appropriate models. The proposed neuroprotective effects should also be discussed separately from observational safety signals reported for long-term PPI exposure, because the evidence types and outcomes are not directly comparable. Pantoprazole’s neurological applications therefore remain experimental pending stronger mechanistic and clinical validation.

### 6.4. Hepatic Fibrosis and Tissue Remodeling

The potential antifibrotic effects of pantoprazole have been investigated in a bile duct ligation-induced liver fibrosis model. Pantoprazole treatment was associated with reduced collagen deposition and hepatic stellate-cell activation, with the reported mechanism involving changes in Yes-associated protein (YAP), a transcriptional regulator implicated in fibrogenesis and tissue remodeling [42]. These findings provide preclinical evidence for a possible effect in this specific model, but they do not establish that YAP is a direct pantoprazole target unless direct target engagement has been demonstrated.

The relevance of this finding to other forms of fibrosis remains uncertain. Fibrosis arising from metabolic, viral, toxic, and cholestatic conditions can involve overlapping but distinct mechanisms. Replication across additional models, exposure-matched mechanistic studies, and assessment of relevant pharmacokinetic and pharmacodynamic parameters are therefore required before a broader antifibrotic role can be proposed.

### 6.5. Iron Homeostasis and Hematological Outcomes

A randomized controlled study in patients with thalassemia major and intermedia reported an association between pantoprazole administration and reduced serum ferritin during standard management [43]. This observation raises the possibility that pantoprazole may influence iron-related physiology in this population. However, serum ferritin is affected by inflammation and other clinical factors and does not independently quantify total tissue iron burden. A reduction in serum ferritin therefore cannot, by itself, establish a clinically meaningful reduction in organ iron deposition or demonstrate a therapeutic benefit.

Confirmation requires replication in appropriately designed studies and assessment using validated measures of iron burden, alongside relevant safety and clinical outcomes. The potential effects of sustained acid suppression on non-haem iron absorption should also be considered, particularly in patients predisposed to iron deficiency. Pantoprazole’s possible effects on iron homeostasis should therefore be interpreted according to the patient population, treatment duration, comparator, and endpoints actually measured.

### 6.6. Integrated Interpretation and Translational Significance

The antimicrobial, metabolic, neurological, hepatic, and hematological literature illustrates the range of biological effects being investigated for pantoprazole; however, the strength and translational maturity of the evidence vary substantially across these domains. The available findings range from cellular and animal experiments to short-term clinical studies and observational data, which should not be regarded as equivalent levels of evidence for therapeutic effectiveness. Although these observations provide a basis for further investigation, they do not establish pantoprazole as an effective treatment for these extra-gastrointestinal conditions.

The evaluation of pantoprazole for repurposing must consider the potential consequences of sustained acid suppression and the limitations of the available safety evidence. Observational studies and meta-analyses have reported associations between PPI use and selected oncological outcomes. Such findings require cautious interpretation because confounding by indication, comorbidity, concomitant medication, surveillance intensity, and reverse causation may influence the observed associations. These data do not, by themselves, establish that pantoprazole causes cancer or worsens cancer prognosis. Conversely, experimental findings suggesting anticancer activity do not establish that pantoprazole protects against cancer or improves survival. Conclusions should therefore distinguish associations from causal evidence and class-level observations from pantoprazole-specific findings.

Sustained suppression of gastric acid can increase circulating gastrin through physiological feedback [83]. Gastrin has trophic effects on selected gastric cell populations, including enterochromaffin-like cells, raising questions about the consequences of prolonged hypergastrinemia in susceptible biological contexts. The clinical significance depends on the magnitude and duration of gastrin elevation, underlying gastric pathology, and the population studied. Hypergastrinemia should therefore be discussed as a biologically plausible consequence of sustained acid suppression rather than as proof that standard pantoprazole treatment promotes malignancy in humans. Where prolonged or high-dose repurposing regimens are proposed, prospective assessment of relevant physiological and safety outcomes is warranted.

Observational studies have also reported associations between PPI exposure and selected neurological outcomes. These associations may be affected by comorbidity, age, concomitant medication, nutritional factors, and other confounders; they should not be interpreted as proof of direct neurotoxicity. Such safety signals must be discussed separately from experimental studies reporting neuroprotective or antiseizure effects of pantoprazole, because these studies address different outcomes and levels of evidence. Neither evidence stream is sufficient on its own to establish the net neurological effect of pantoprazole in humans.

Iron homeostasis is another relevant consideration. Gastric acid contributes to the solubilization and absorption of non-haem iron, so sustained acid suppression may be relevant in patients with iron deficiency or impaired iron absorption [84]. At the same time, reports of reduced serum ferritin in selected clinical populations require careful interpretation because ferritin is influenced by inflammation and other clinical factors and does not independently quantify tissue iron burden. Changes in ferritin should not automatically be interpreted as a clinically meaningful reduction in organ iron deposition. Further studies should use appropriate iron-related endpoints and account for the underlying disease, treatment regimen, and duration of exposure.

Overall, the relative drug-interaction advantage of pantoprazole should not be conflated with an absence of adverse effects or with proof of efficacy for new indications. High-dose intravenous administration, prolonged treatment, and combination regimens require specific assessment of pharmacokinetics, pharmacodynamics, and safety. The safety evidence obtained during routine acid-related treatment cannot automatically establish the safety of materially different repurposed regimens. Future research should prioritize exposure-matched mechanistic studies, independent replication in relevant experimental models, and appropriately designed clinical trials to determine whether the reported effects are reproducible, clinically meaningful, and achievable at safe therapeutic exposures.

## 7. Translational Challenges and Future Directions

The expanding literature on pantoprazole raises an important pharmacological question: under what conditions can a clinically established drug produce reproducible and clinically meaningful effects beyond its approved gastrointestinal indications? Addressing this question requires a translational framework connecting drug exposure, molecular mechanism, target engagement, pharmacodynamic response, disease-relevant phenotype, safety, and clinical outcome. Evidence at one stage of this sequence cannot automatically establish the next. Cellular pathway modulation may identify a biological hypothesis, but its relevance depends on the experimental concentration, duration of exposure, and specificity of the response. Animal efficacy provides additional evidence, although its interpretation depends on model validity, pharmacokinetic relevance, and mechanistic confirmation. Human observational findings can generate clinically relevant hypotheses but remain vulnerable to confounding and indication bias. Ultimately, the translational value of pantoprazole depends on whether its effects can be connected quantitatively and mechanistically from exposure to target modulation and then to reproducible, patient-relevant outcomes.

One of the most important unresolved issues is the relationship between experimental concentration and clinically achievable exposure. Several anticancer studies have reported substantial biological activity at concentrations that may exceed those routinely achieved in human plasma during conventional pantoprazole therapy [44,47,74,75]. These findings may remain valuable for identifying potential mechanisms, but they cannot be assumed to predict clinical activity without further investigation. Studies should determine whether pantoprazole or relevant metabolites reach the target tissue at adequate concentrations and for sufficient duration to produce the proposed response. This requires consideration of total and unbound plasma concentrations, tissue distribution, local drug accumulation, and, where relevant, activation kinetics. Experiments should establish exposure–response relationships and link measured exposure to specific pharmacodynamic endpoints. Continuous exposure in cell culture should not be equated with the transient systemic exposure following routine oral administration, and animal doses should not be translated into human efficacy solely by nominal dose conversion.

In addition to the exposure–mechanism gap, target specificity remains an important unresolved issue. Although studies report effects on several signaling pathways, these findings do not necessarily indicate direct molecular interactions between pantoprazole and each pathway component. Changes in autophagy, V-ATPase-associated processes, TOPK, STAT3, YAP, or drug-efflux transporters may represent direct effects in some experimental settings or downstream consequences of a more proximal perturbation. The earliest reproducible molecular event should therefore be identified using orthogonal approaches, including biochemical assays, target-engagement studies, pharmacological controls, genetic perturbation, and rescue experiments where appropriate. These experiments should be performed at concentrations relevant to the intended clinical application. For anticancer studies in particular, pharmacodynamic endpoints are essential to distinguish a failure to achieve adequate exposure or target engagement from a failure of the proposed biological mechanism itself.

An important issue for future research is the distinction between pantoprazole-specific effects and effects shared across the PPI class. Studies that investigate PPIs collectively or focus on another member of the class cannot automatically establish a pantoprazole-specific mechanism. Comparative studies should assess individual PPIs using appropriate controls and, where feasible, comparable exposure conditions. Such comparisons should examine pharmacokinetics, drug-interaction potential, target engagement, biological activity, and safety. Pantoprazole’s comparatively favorable CYP2C19-mediated interaction profile relative to omeprazole and esomeprazole may be relevant when selecting a candidate for combination treatment, but it does not independently establish superior activity for a repurposed indication. A reproducible advantage over other PPIs for a defined biological endpoint would provide a stronger rationale for drug-specific development, whereas evidence of a class effect would support a different development strategy.

Combination therapy represents another potentially productive direction for future development. The available evidence repeatedly suggests that pantoprazole may function more effectively as a sensitizer, modifier, or pharmacological adjunct than as a stand-alone treatment for several proposed indications. In oncology, this concept includes combinations with chemotherapy and vitamin C [45,47,48], whereas in antimicrobial disease the proposed role is primarily to enhance conventional antimicrobial activity [9,31,33,34,35]. A repurposed drug often provides a new mechanism of action that can modify resistance, cellular stress, drug accumulation or treatment sensitivity in addition to, or even instead of directly treating the disease. Thus, a repurposed drug does not need to be as effective as the conventional disease-specific treatment to provide clinical value. Indeed, its greatest value may lie in its ability to make cells or microbes already diseased more susceptible to established therapies. However, as with any therapy, the pharmacological interactions between repurposed and established drugs may be synergistic, additive, or antagonistic, increasing toxicity as well as potential therapeutic benefit. Therefore, formal combination trials incorporating mechanistic pharmacodynamic studies are necessary to assess the true potential of repurposed drugs, distinguish synergy from simple additive effects, and determine whether interactions persist at clinically relevant drug exposures. Patient selection based on molecular or other relevant characteristics will be particularly important for identifying clinically meaningful effects.

This principle is exemplified by the PANDORA trial, which evaluated pantoprazole in combination with docetaxel for the treatment of prostate cancer based on preclinical evidence suggesting that inhibition of autophagy enhances the antitumor activity of this conventional chemotherapy drug [49]. The PANDORA phase II study illustrates the difficulty of translating preclinical findings into clinical benefit. Twenty-one men with metastatic castration-resistant prostate cancer received intravenous pantoprazole at 240 mg before docetaxel 75 mg/m^2^ every 21 days, together with continuous prednisone. A prostate-specific antigen (PSA) response occurred in 11 of 21 patients (52%), but the prespecified threshold of at least 13 responders was not met, and the trial did not proceed to its second stage [49]. The regimen was reported to be tolerable, but its clinical activity was insufficient to meet the predefined criterion for further testing. These findings reinforce the need for adequate exposure, demonstrable target engagement, appropriate patient selection, and prospectively defined clinical endpoints when evaluating pantoprazole as a repurposed agent. Factors that might explain the limited clinical activity of a highly plausible preclinical concept include (i) insufficient target modulation at clinically tolerated doses; (ii) inadequate exposure of the experimental drug to the tumor; (iii) inappropriate selection of patients; (iv) high degree of biological heterogeneity of the target; (v) suboptimal drug combinations; and (vi) lack of persistence or dominance of the experimental mechanism of action over other pathways within tumors. Therefore, the PANDORA trial provides an important proof of concept regarding the need for mechanistically driven preclinical studies to be coupled with rigorous pharmacological validation in human tumors. Negative studies should also provide evidence regarding the extent to which the intended target was modulated in patients.

Similarly, considerations with respect to safety must be refined when a PPI is to be investigated for use outside of its approved indications. Safety data for established PPIs have been extensively collected in the many patients treated for gastrointestinal disease. The physiological effects of long-term PPI treatment such as low grades of hypomagnesemia and alterations in the gastrointestinal microbiota have been characterized and are generally transient [4,5,80]. However, such data were generally collected in short term studies and at lower doses than those used in some contemporary settings and must be re-evaluated in the light of the specific treatment schedule (dose and duration) as well as patient population intended for repurposing of pantoprazole. Importantly, these evaluations should be performed within the framework of the intended mechanism of action (therapeutic effect) and may require doses and exposure levels that are different from those required for its established use as an acid inhibitor. Evaluation of adverse effects should, in addition to effects appearing early in treatment, also include consideration of possible late-appearing, i.e., cumulative, effects and potential for effects on micronutrient homeostasis, gastrointestinal ecology, and effects leading to drug interactions, as well as other related effects that could become problematic during long-term treatment of patients.

An additional priority is the development of biomarkers capable of identifying patients most likely to benefit from pantoprazole-based interventions. The broad range of biological effects described in experimental studies suggests that pantoprazole is unlikely to exert uniform therapeutic effects across all disease states or patient populations. Instead, its activity may depend on the metabolic phenotype of the target tissue, intracellular proton-handling capacity, dependence on autophagy or specific survival pathways, inflammatory status, oxidative stress burden or expression of relevant transport and signaling proteins. Biomarker-guided investigation could therefore convert the current broad concept of pleiotropy into a more precise framework of context-dependent pharmacology. Rather than asking whether pantoprazole is effective across an entire disease category, future studies should determine whether a biologically defined subgroup demonstrates target engagement and enhanced sensitivity to pantoprazole-containing therapy. This approach would be particularly relevant to oncology, where molecular heterogeneity can obscure meaningful treatment effects when mechanistically distinct tumors are analyzed together. The same principle may be applicable to inflammatory, infectious and metabolic disorders, in which disease heterogeneity may determine whether modulation of proton handling, oxidative stress, inflammatory signaling or metabolic pathways produces a clinically relevant response.

**Figure 2:**
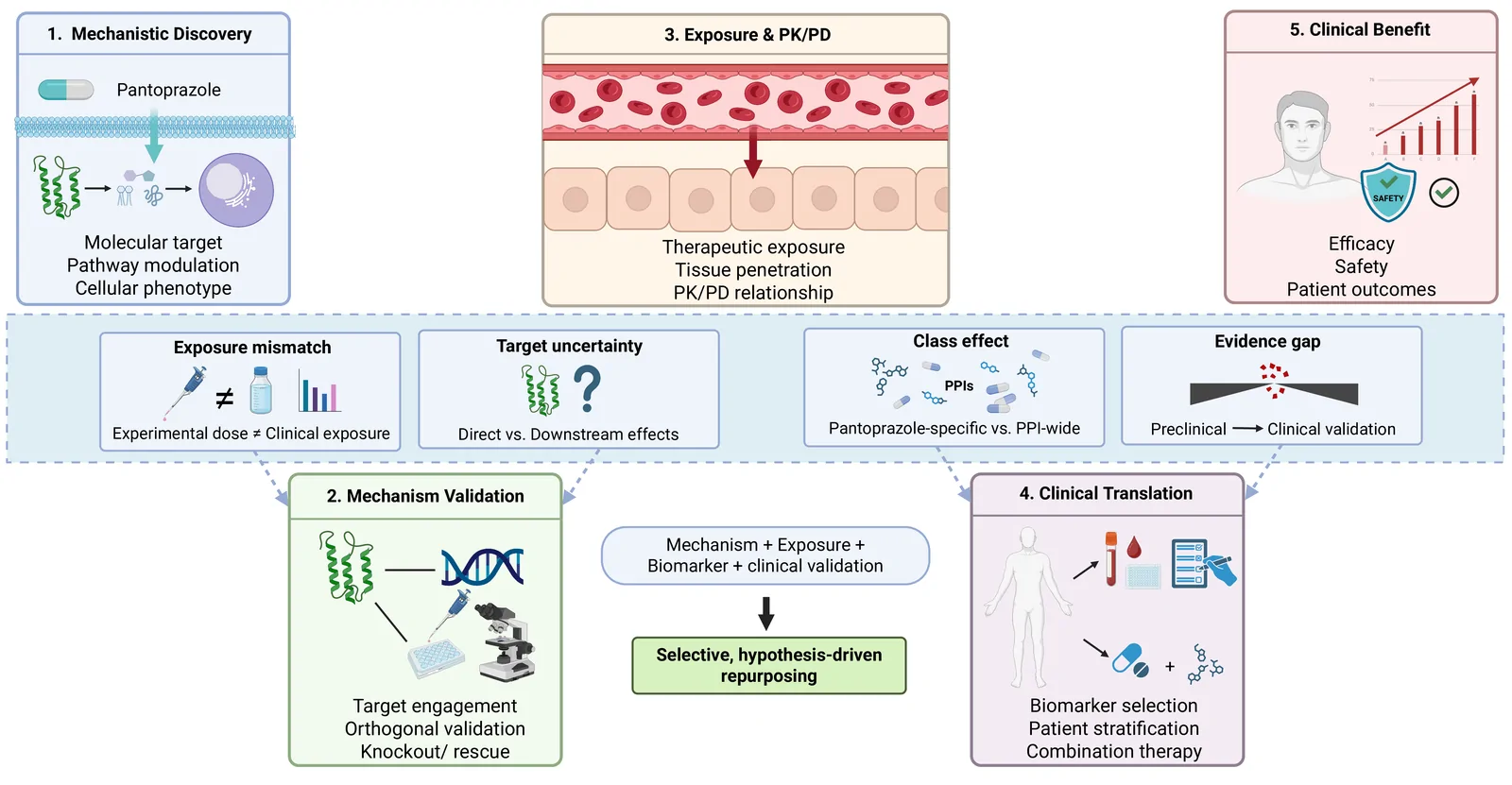
Translational framework for hypothesis-driven repurposing of pantoprazole. This diagram presents a stepwise framework for validating central stress-modulating effects of pantoprazole into clinically meaningful applications. Created in BioRender. Satyam, D.S.M. (2026) https://BioRender.com/9ng56nc. Abbreviations: PD, pharmacodynamics; PK, pharmacokinetics; PK/PD, pharmacokinetic/pharmacodynamic; PPIs, proton pump inhibitors.

Taken together, the available evidence supports a conceptual model in which pantoprazole can influence a network of biological processes centred on proton homeostasis, cellular stress adaptation, organelle function and downstream survival signaling. In malignant cells, these effects may reduce the capacity to maintain an adaptive phenotype under metabolic or therapeutic stress. In inflammatory and ischemic tissues, they may attenuate interactions among oxidative injury, inflammatory signaling and apoptosis. In microorganisms, they may interfere with proton-dependent stress tolerance or resistance-associated mechanisms. In other tissues, the same broad pharmacological properties may manifest as changes in metabolic regulation, fibrosis, neuronal stress or iron metabolism. The apparent diversity of these phenotypes should not automatically be interpreted as evidence of nonspecific pharmacological effects, nor should it be assumed to arise from a single shared molecular mechanism. Instead, these effects may reflect context-dependent responses to the modulation of interconnected cellular pathways. However, this proposed convergence remains a hypothesis-generating framework rather than definitive evidence of a common pleiotropic mechanism. Future work should establish which effects share a common molecular origin and which are independent or secondary pharmacologic consequences. This distinction is crucial to advancing research on pantoprazole from descriptive pleiotropy to testable pharmacologic hypotheses (Figure 2).

Hence, the next step in pantoprazole investigation should switch from the random search of additional indications to mechanistic, translational validation. The initial objective should be to establish clinically achievable exposure, identify the relevant primary molecular target, demonstrate target engagement in disease-relevant tissue, define pharmacodynamic and predictive biomarkers, differentiate responders from non-responders, and evaluate rational combination strategies. Studies should be designed prospectively around a defined molecular dependency for which experimental evidence exists, rather than on the broad assumption that pantoprazole will exert nonspecific protective or cytotoxic effects. In settings where there is already some evidence of activity in humans, adequately powered randomized clinical trials should determine whether these biological effects translate into meaningful improvements in survival, disease control, organ preservation, symptom burden or other clinically relevant outcomes. Equally important is the mechanistic interpretation of negative findings. In the case of failure of efficacy despite confirmed target engagement, the therapeutic hypothesis itself would be challenged, whereas failure without target engagement would mostly indicate insufficient pharmacological delivery, exposure or patient selection. This distinction should become a guiding principle for future pantoprazole repurposing studies, since it reveals whether a negative result closes a therapeutic avenue or rather points to a modifiable pharmacological problem.

## 8. Conclusions

Pantoprazole is an established acid-suppressive medicine with a well-characterized pharmacological role and a comparatively favorable CYP2C19-mediated drug-interaction profile relative to omeprazole and esomeprazole. These properties provide a rationale for continued investigation of selected repurposing hypotheses. Experimental studies have reported effects involving cellular proton homeostasis, autophagy, apoptosis, oxidative and inflammatory signaling, antimicrobial resistance, glucose-related pathways, hepatic tissue remodeling, and iron-related outcomes. However, the evidence varies substantially in mechanistic depth, experimental quality, exposure relevance, and clinical validation.

A central unresolved question is whether the effects reported in experimental systems can be reproduced at clinically relevant exposures in the appropriate human tissues. Acid-dependent activation, local drug accumulation, target engagement, and the difference between sustained in vitro exposure and transient systemic exposure must be considered when interpreting extragastric findings. In addition, downstream pathway changes should not be presented as proof of direct molecular targeting unless supported by appropriate experimental evidence. The available clinical findings, including the limitations illustrated by the PANDORA study, reinforce the need to distinguish biological plausibility and tolerability from demonstrated clinical benefit.

Pantoprazole’s relatively low propensity for CYP2C19-mediated interactions compared with omeprazole and esomeprazole may be relevant when considering combination treatment, but this advantage does not establish efficacy for a new indication or eliminate all interaction and safety concerns. Potential consequences of sustained acid suppression should also be considered when designing prolonged or high-dose regimens. Overall, pantoprazole should currently be regarded as a candidate for mechanism-informed drug-repurposing research rather than an established treatment for the non-gastrointestinal indications discussed in this review. Exposure-matched mechanistic studies, pharmacokinetic and pharmacodynamic validation, reproducible preclinical evidence, and appropriately designed clinical trials are required to determine whether any of these proposed effects translate into clinically meaningful benefit.

## Figures and Tables

**Table 1:** Representative experimental and clinical studies of pantoprazole: models, exposure parameters, principal findings, and translational relevance.

Study Title and Reference	Model or Study Population	Principal Endpoint or Finding	Evidence Level and Translational Limitation
The proton pump inhibitor pantoprazole disrupts protein degradation systems and sensitizes cancer cells to death under various stresses [7]	Cancer-cell models under cellular stress	Disruption of protein-degradation systems and increased cancer-cell sensitivity to stress	In vitro mechanistic/preclinical evidence; clinical relevance depends on exposure and target engagement
Up-regulation of autophagy is a mechanism of resistance to chemotherapy and can be inhibited by pantoprazole to increase drug sensitivity [14]	Experimental cancer and chemotherapy-resistance models	Inhibition of autophagy-associated resistance and increased drug sensitivity	Mechanistic/preclinical evidence; does not establish clinical benefit
Effect of pantoprazole on IR-induced myocardial injury in diabetic rats targeting inflammatory cytokine release and oxidative stress [19]	Diabetic rats with myocardial ischemia–reperfusion injury	Changes in inflammatory cytokines and oxidative-stress-associated myocardial injury	Animal evidence; human dose equivalence and clinical benefit are unestablished
Pantoprazole attenuates MAPK (ERK1/2, JNK, p38)–NF-κB and apoptosis signaling pathways after renal ischemia/reperfusion injury in rats [20]	Rat renal ischemia–reperfusion model	Modulation of MAPK/NF-κB signaling and apoptosis-associated endpoints	Animal evidence; pathway modulation does not alone establish a direct molecular target
The protective effect of acute pantoprazole pretreatment on renal ischemia/reperfusion injury in rats [21]	Rat renal ischemia–reperfusion model	Renal injury and associated protective endpoints	Animal evidence; acute pretreatment may not represent sustained human treatment
Pantoprazole abrogated cisplatin-induced nephrotoxicity in mice via suppression of inflammation, apoptosis, and oxidative stress [22]	Mice with cisplatin-induced nephrotoxicity	Inflammatory, oxidative-stress, apoptotic, and renal-injury endpoints	Animal evidence; protective effects must be assessed alongside any effect on anticancer efficacy
Uroprotective effect of pantoprazole against cyclophosphamide-induced cystitis in mice [23]	Mouse model of cyclophosphamide-induced cystitis	Bladder injury and associated oxidative/inflammatory endpoints	Animal evidence; clinical uroprotection remains unestablished
Protective effect of pantoprazole against sepsis-induced acute lung and kidney injury in rats [24]	Rat model of sepsis-associated organ injury	Lung and kidney injury, inflammatory mediators, and proposed RhoA/ROCK-related signaling	Animal evidence; does not establish effectiveness in human sepsis
Redirecting pantoprazole as a metallo-beta-lactamase inhibitor in carbapenem-resistant *Klebsiella pneumoniae* [31]	Bacterial and enzyme-based experimental systems	Metallo-β-lactamase inhibition and potential modification of carbapenem resistance	In vitro evidence; clinical relevance depends on achievable infection-site exposure and validated antimicrobial outcomes
The effect of hypergastrinemia following sleeve gastrectomy and pantoprazole on type 2 diabetes mellitus and beta-cell mass in Goto-Kakizaki rats [36]	Goto–Kakizaki diabetic rats	Gastrin levels, glucose metabolism, and pancreatic β-cell outcomes	Animal evidence; does not establish clinically meaningful glucose lowering in humans
Study of the effect of pantoprazole on glycemic control of type-2 diabetes mellitus in tertiary care center and hospital in North India [37]	Patients with type 2 diabetes mellitus	Glycemic-control outcomes	Human clinical evidence; interpret in light of study design, duration, and possible confounding
The effects of proton pump inhibitors (pantoprazole) on pentylenetetrazole-induced epileptic seizures in rats and neurotoxicity in the SH-SY5Y human neuroblastoma cell line [41]	Rat seizure model and SH-SY5Y cells	Seizure-associated outcomes and cellular neurotoxicity/stress endpoints	Mixed animal and in vitro evidence; does not establish human neuroprotection or antiseizure efficacy
Pantoprazole ameliorates liver fibrosis and suppresses hepatic stellate cell activation in bile duct ligation rats by promoting YAP degradation [42]	Rat bile duct ligation model of liver fibrosis	Collagen deposition, hepatic stellate-cell activation, and YAP-associated findings	Animal evidence; YAP should not be described as a direct target without direct target-engagement evidence
Eghbali et al. (2019), Pantoprazole reduces serum ferritin in patients with thalassemia major and intermedia: A randomized, controlled study [43]	Patients with thalassemia major or intermedia	Change in serum ferritin	Human randomized evidence; serum ferritin alone does not establish a reduction in organ iron burden
Pantoprazole, an FDA-approved proton-pump inhibitor, suppresses colorectal cancer growth by targeting T-cell-originated protein kinase [44]	Colorectal cancer experimental models	TOPK-associated effects and experimental colorectal tumour growth	Mechanistic and preclinical evidence; clinical relevance depends on exposure and target engagement
Proton pump inhibitors increase the chemosensitivity of patients with advanced colorectal cancer [45]	Patients with advanced colorectal cancer; consult the original article for any accompanying experimental models	Reported association between PPI use and chemosensitivity	Human clinical/observational evidence as applicable to the study design; cannot automatically establish a pantoprazole-specific causal effect
Pantoprazole attenuates tumorigenesis via inhibition of exosomal secretion in a rat model of hepatic precancerous lesion induced by diethylnitrosamine and 2-acetamidofluorene [46]	Rat model of chemically induced hepatic precancerous lesions	Precancerous lesions and extracellular-vesicle/exosome-associated outcomes	Animal evidence; does not establish prevention of human liver cancer
Pantoprazole pretreatment elevates sensitivity to vincristine in drug-resistant oral epidermoid carcinoma in vitro and in vivo [47]	Vincristine-resistant oral epidermoid carcinoma models	Vincristine sensitivity and P-glycoprotein-associated drug accumulation	In vitro and animal evidence; does not establish clinical chemosensitization
Drug repurposing of pantoprazole and vitamin C targeting tumor microenvironment conditions improves anticancer effect in metastatic castration-resistant prostate cancer [48]	Prostate cancer experimental models, including xenograft models	Combination-associated effects on tumour-cell activity and xenograft growth	Combination preclinical evidence; cannot be attributed to pantoprazole alone
Pantoprazole affecting docetaxel resistance pathways via autophagy (PANDORA): phase II trial of high dose pantoprazole with docetaxel in metastatic castration-resistant prostate cancer [49]	Patients with metastatic castration-resistant prostate cancer	Clinical response and whether the prespecified criterion for continuation was met	Human phase II evidence; the study did not meet its prespecified criterion for proceeding to the second stage

Abbreviations: IR, ischemia–reperfusion; MAPK, mitogen-activated protein kinase; NF-κB, nuclear factor kappa B; PPI, proton pump inhibitor; RhoA/ROCK, Ras homolog family member A/Rho-associated coiled-coil-containing protein kinase; TOPK, T-cell-originated protein kinase; YAP, Yes-associated protein.

## Data Availability

No new data were created or analyzed in this study. Data sharing is not applicable to this article.

## Abbreviations

The following abbreviations are used in this manuscript:

Abbreviation	Full term
ABCG2	ATP-binding cassette subfamily G member 2
Bcl-2	B-cell lymphoma 2
Bax	Bcl-2-associated X protein
CC BY	Creative Commons Attribution
CD24	Cluster of differentiation 24
CD44	Cluster of differentiation 44
CYP2C19	Cytochrome P450 family 2 subfamily C member 19
EMT	Epithelial–mesenchymal transition
EpCAM	Epithelial cell adhesion molecule
EV	Extracellular vesicle
FOXM1	Forkhead box M1
H^+^/K^+^-ATPase	Hydrogen/potassium adenosine triphosphatase
IL-1β	Interleukin-1 beta
IL-6	Interleukin-6
Lgr5	Leucine-rich repeat-containing G-protein-coupled receptor 5
MAPK	Mitogen-activated protein kinase
NF-κB	Nuclear factor kappa B
PDA	Pancreatic ductal adenocarcinoma
PI3K/Akt/mTOR	Phosphoinositide 3-kinase/protein kinase B/mechanistic target of rapamycin
PPIs	Proton pump inhibitors
ROS	Reactive oxygen species
RhoA/ROCK	Ras homolog family member A/Rho-associated coiled-coil-containing protein kinase
SHP-1	Src homology region 2 domain-containing phosphatase-1
STAT3	Signal transducer and activator of transcription 3
TNF-α	Tumor necrosis factor-alpha
TOPK	T-cell-originated protein kinase
V-ATPase	Vacuolar-type H^+^-ATPase
Wnt/β-catenin	Wingless-related integration site/β-catenin
YAP	Yes-associated protein
